# Supplementary material for: Intestinal Barrier Permeability in Obese Individuals with or without Metabolic Syndrome: A Systematic Review
Source: Nutrients. 2022 Sep 3;14(17):3649. doi: 10.3390/nu14173649 (PMC9459697; doi:10.3390/nu14173649)
Supplement: Supplementary file 1 [file nutrients-14-03649-s001.zip › File S1 appendix.pdf]

File S1. Full electronic search strategy for PubMed, ScienceDirect, Scopus, LILACS and EMBASE databases

PubMed and ScienceDirect

(“intestinal absorption” OR “intestinal permeability”) AND (obesity OR overweight) AND “metabolic syndrome”

(“intestinal absorption” OR “intestinal permeability”) AND (obesity OR overweight) NOT “metabolic syndrome”

Scopus

TITLE-ABS-KEY (“intestinal absorption” OR “intestinal permeability”) AND TITLE-ABS-KEY (obesity OR overweight) AND TITLE-ABS-KEY “metabolic syndrome”

TITLE-ABS-KEY (“intestinal absorption” OR “intestinal permeability”) AND TITLE-ABS-KEY (obesity OR overweight) AND TITLE-ABS-KEY “metabolic syndrome”

Limitations: Portuguese OR English OR Spanish

LILACS

(“permeabilidade intestinal” OR “função da barreira intestinal” OR “barreira intestinal”) AND obesidade AND “síndrome metabólica”

(“permeabilidade intestinal” OR “função da barreira intestinal” OR “barreira intestinal”) AND obesidade

Embase

(‘intestinal permeability’ OR ‘intestine absorption’) AND obesity AND ‘metabolic syndrome X’

(‘intestinal permeability’ OR ‘intestine absorption’) AND obesity NOT ‘metabolic syndrome X’

The search was commenced in August 2020 and the last update was performed on December 2021.
