# Supplementary figures and images for: Intestinal Barrier Permeability in Obese Individuals with or without Metabolic Syndrome: A Systematic Review
Source: Nutrients. 2022 Sep 3;14(17):3649. doi: 10.3390/nu14173649 (PMC9459697; doi:10.3390/nu14173649)

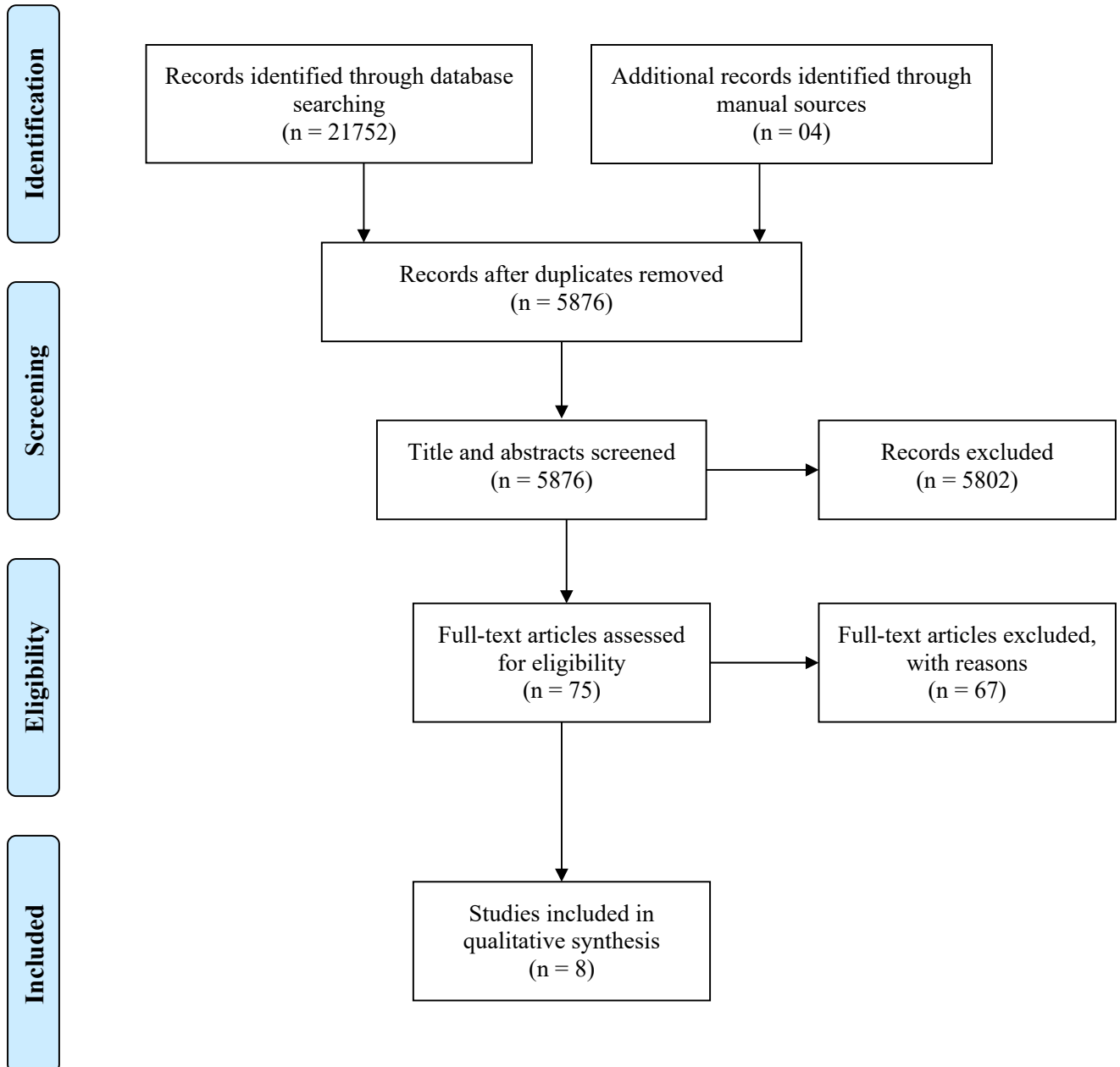

Supplement: Supplementary file 1 [file nutrients-14-03649-s001.zip › Figure S1 PRISMA flow diagram.pdf]
